# Role of IRE1α/XBP1/CHOP/NLRP3 Signalling Pathway in Neonicotinoid Imidacloprid-Induced Pancreatic Dysfunction in Rats and Antagonism of Lycopene: In Vivo and Molecular Docking Simulation Approaches

**DOI:** 10.3390/toxics12070445

**Published:** 2024-06-21

**Authors:** Walaa Bayoumie El Gazzar, Heba Bayoumi, Heba S. Youssef, Tayseer A. Ibrahim, Reham M. Abdelfatah, Noha M. Gamil, Mervat K. Iskandar, Amal M. Abdel-Kareim, Shaymaa M. Abdelrahman, Mohammed A. Gebba, Mona Atya Mohamed, Maha M. Mokhtar, Tayseir G. Kharboush, Nervana M. Bayoumy, Hatun A. Alomar, Amina A. Farag

**Affiliations:** 1 Department of Anatomy, Physiology and Biochemistry, Faculty of Medicine, The Hashemite University, P.O. Box 330127, Zarqa 13133, Jordan; wallagazzar@hu.edu.jo; 2 Department of Medical Biochemistry & Molecular Biology, Faculty of Medicine, Benha University, Benha 13518, Egypt; shaimaa.bghdadi@fmed.bu.edu.eg; 3 Department of Histology and Cell Biology, Faculty of Medicine, Benha University, Benha 13518, Egypt; heba.bayoumi@fmed.bu.edu.eg (H.B.); mona.fady@fmed.bu.edu.eg (M.A.M.); 4 Department of Physiology, Faculty of Medicine, Benha University, Benha 13518, Egypt; heba.youssef@fmed.bu.edu.eg (H.S.Y.); tayseer.alaa@fmed.bu.edu.eg (T.A.I.); 5 Department of Pesticides, Faculty of Agriculture, Mansoura University, Mansoura 35516, Egypt; reham_2010@mans.edu.eg; 6 Department of Pharmacology and Toxicology, Faculty of Pharmaceutical Sciences and Drug Manufacturing, Misr University for Science and Technology, 6th of October City 12573, Egypt; noha.mohsen@must.edu.eg; 7 Department of Zoology, Faculty of Science, Benha University, Benha 13518, Egypt; mervat.iskandar@fsc.bu.edu.eg (M.K.I.); amel.abdelkarim@fsc.bu.edu.eg (A.M.A.-K.); 8 Department of Anatomy& Embryology, Faculty of Medicine, Benha University, Benha 13518, Egypt; mohamed.gaba@fmed.bu.edu.eg; 9 Department of Forensic Medicine and Clinical Toxicology, Faculty of Medicine, Benha University, Benha 13518, Egypt; maha.mahmoud@fmed.bu.edu.eg; 10 Department of Pharmacology and Therapeutics, Faculty of Medicine, Benha University, Benha 13518, Egypt; tyseerg40@gmail.com; 11 Department of Physiology, College of Medicine, King Saud University, Riyadh 11461, Saudi Arabia; nbayoumy@ksu.edu.sa; 12 Pharmacology and Toxicology Department, Faculty of Pharmacy, King Saud University, Riyadh 11451, Saudi Arabia; hetalomar@ksu.edu.sa

**Keywords:** imidacloprid, lycopene, endoplasmic reticulum stress, apoptosis, pyroptosis, pancreas

## Abstract

Imidacloprid (IMI) is a commonly used new-generation pesticide that has numerous harmful effects on non-targeted organisms, including animals. This study analysed both the adverse effects on the pancreas following oral consumption of imidacloprid neonicotinoids (45 mg/kg daily for 30 days) and the potential protective effects of lycopene (LYC) administration (10 mg/kg/day for 30 days) with IMI exposure in male Sprague–Dawley rats. The apoptotic, pyroptotic, inflammatory, oxidative stress, and endoplasmic reticulum stress biomarkers were evaluated, along with the histopathological alterations. Upon IMI administration, noticeable changes were observed in pancreatic histopathology. Additionally, elevated oxidative/endoplasmic reticulum-associated stress biomarkers, inflammatory, pyroptotic, and apoptotic biomarkers were also observed following IMI administration. LYC effectively reversed these alterations by reducing oxidative stress markers (e.g., MDA) and enhancing antioxidant enzymes (SOD, CAT). It downregulated ER stress markers (IRE1α, XBP1, CHOP), decreased pro-inflammatory cytokines (TNF-α, IL-1β), and suppressed pyroptotic (NLRP3, caspase-1) along with apoptotic markers (Bax, cleaved caspase-3). It also improved the histopathological and ultrastructure alterations brought on by IMI toxicity.

## 1. Introduction

Numerous xenobiotics pose serious risks to both people and the environment. The most prevalent pollutants, pesticides, affect biological structures in both acute and chronic exposures in various ways [1]. Over the past few decades, neonicotinoid pesticides have experienced the fastest rate of growth [2]. Neonicotinoids are frequently used due to their high toxicity to invertebrates, simplicity, versatility, long-lasting effects, and systemic distribution throughout the target crop [3]. However, these characteristics increase the risk of environmental contamination and adverse health effects on exposed organisms. These substances are widely distributed throughout the ecosystem [4], making it easier to encounter toxic levels through inhalation, skin contact, and ingestion of contaminated produce and water [2,5]. IMI is a new-generation pesticide suggested as a safer alternative [4], but its extensive use has been associated with various side effects on non-targeted species [3]. Zhao et al., who reported the presence of IMI and its metabolites in urine samples of the general population, demonstrated this [2,4]. Accumulating research on mammals, chicken embryos, and honey bees suggests that IMI, in addition to causing gastrointestinal insults and neurological symptoms, leads to renal damage, as indicated by increased activity of glutamate pyruvate transaminase and glutamate oxalacetate transaminase, elevated glucose and blood urea nitrogen content, cardiovascular insults, haematological impairments, renal and reproductive organ damage, and hepatotoxicity, reflected in increased bilirubin levels and alterations in liver tissue [3,4,5]. Recently, IMI’s toxic effects on the pancreas have been observed. Findings suggest that IMI may contribute to type 2 diabetes mellitus (T2DM), one of the major metabolic disorders characterized by pancreatic β-cell dysfunction. In addition to one of the diabetogenic pesticides [1], we focus on the pancreas, an organ highly susceptible to oxidative stress and inflammation, particularly inflammatory mediators that affect vascular permeability (e.g., TNF-α, IL-1β, and IL-6) [6], resulting in acinar and β cell dysfunction [7].

According to Karpińska and Czauderna (2022), emerging pancreatic insufficiency is the result of damage to the exocrine region of the pancreas, which causes the pancreas to be unable to biosynthesize and/or produce enough digestive enzymes for the intestines to process and absorb food parts. In addition, diabetes mellitus and its consequences on varied systems of the body develop from beta cell damage [2,3].

Various studies have suggested that the main mechanism of IMI toxicity is oxidative stress combined with inflammation [6,8]. Recent experiments have shown that the NOD-like receptor family pyrin domain-containing 3 (NLRP3) inflammasome plays a crucial role in linking β cell dysfunction and inflammation through its pyroptotic function, which leads to cellular swelling, plasma membrane rupture, and secretion of interleukin (IL)-1β [9]. Although IMI has been observed to induce cellular inflammation, the specific mechanism of IMI-induced pancreatic pyroptosis remains unclear. Tumour necrosis factor alpha (TNF-α) is a vital component in stimulating the NLRP3 inflammasome, and studies have demonstrated its significant exacerbation by IMI-induced oxidative stress [10,11,12]. TNF-α is also necessary for the activation of caspase-1 [13]. Currently, there is limited understanding of the mechanism underlying IMI-induced TNF-α-associated cellular damage, and thus, further research is needed to determine whether TNF-α mediates IMI-induced inflammasome production and pyroptosis.

In the presence of abnormally folded or unfolded proteins, the endoplasmic reticulum (ER) directs these proteins to the cytoplasm, where they are eliminated via an ER-associated degradation process (ERAD) [4]. However, in states like inflammation and oxidative stress, the ER may not be able to cope with the levels of damaged proteins, thus triggering ER stress and the unfolded protein response (UPR), which in turn activates the NLRP3 inflammasome, causing cell pyroptosis [4,5,6,8]. This process has been associated with neurodegenerative, cardiovascular, and metabolic diseases, such as diabetes mellitus. Although a few studies have demonstrated an association between increased ER stress and IMI exposure, the relationship remains unclear and requires further investigation to comprehensively understand the process and develop innovative treatment methods for IMI toxicity [13].

Many studies have established a link between ER stress and NLRP3-mediated pyroptosis in the development of pancreatic damage. If research can develop a method to alleviate this effect, it may serve as a therapeutic approach to reduce IMI-induced pancreatic damage [14].

Lycopene, a non-provitamin A carotenoid, is abundant in the human diet. It is not synthesized by the body and can only be obtained through dietary consumption [15]. According to Milani et al., lycopene exhibits antioxidant properties by scavenging O2 and OH-, thereby reducing the risk of oxidative stress-related chronic illnesses such as cancer, hypertension, cardiovascular disease, and pancreatic disorders [16,17]. Furthermore, it inhibits oxidative stress and inflammation by suppressing the NF-κB pathway [18]. However, its protective effect on the pancreas against IMI has not been studied extensively.

This study aims to elucidate the protective effects of lycopene on various cellular activities and signalling pathways, as well as to investigate its interaction with the pathogenesis of IMI-induced pancreatic dysfunction and to review the literature.

## 2. Materials and Methods

### 2.1. Drugs and Chemicals

Imidacloprid (IMI) [1-(6-chloro-3-pyridylmethyl)-N-nitroimidazolidin-2-ylideneamine] [CAS No.138261-41-3] was obtained from BAYER company, Leverkusen, Germany. Lycopene (LYC) (C40H56; deep red powder CAS Number 502–65-8, purity > 95%) was obtained from Sigma-alders, St. Louis, MO, USA.

### 2.2. Animals and Ethics

Twenty-eight rats were utilized in this experiment. The general characteristics of the animals were as follows: (1) the rats were eight weeks old, (2) their weight ranged between 150 and 180 g (166.21 ± 10.82), and (3) they were male Sprague–Dawley rats. The rats were obtained from the animal house colony of the Faculty of Science, Benha University, Egypt. Throughout the entire experiment, the rats were divided into four groups and housed in well-ventilated cages at a room temperature of 23  ±  2 °C, with a relative humidity of 55  ±  5% and a 12 h light/dark cycle. The animals had ad libitum access to water as a source of food/fluid. The Research Ethics Committee of Benha University Faculty of Science (BUFS-REC-2023-34 Zoo) approved all animal interventions and conditions, complying with the Guidelines of the Laboratory Animals (NIH Publication No. 8023, revised).

### 2.3. Experimental Design

The rats were randomly assigned to four groups, each consisting of seven rats, as follows:

- Group I: Rats received 0.5 mL/day of corn oil, which served as the vehicle used in preparing IMI and LYC.
- Group II: Rats were treated with LYC dissolved in 0.5 mL corn oil at a dose of 10 mg/kg/day. Previous investigations have demonstrated that this dose is effective in producing antioxidant defence and reducing the toxicity of certain xenobiotics [19,20,21,22,23,24].
- Group III: Rats were treated with IMI at a dose of 45 mg/kg, which is 1/10 of the LD50 [25,26,27].
- Group IV: Rats were treated with a combination of IMI (45 mg/kg) and LYC (10 mg/kg).

All drugs were administered orally on a daily basis for a duration of 30 days. Rats were put under anaesthesia using isoflurane. Using a cardiac puncture, blood samples were collected in plain sample bottles, and a laparotomy was conducted to obtain samples of pancreatic tissue. Blood samples were centrifuged (3000× *g* for a duration of 10 min) at room temperature, and the separated serum was used to analyse pancreatic enzymes and insulin levels. During the laparotomy, the pancreas was separated and dissected into three parts (head, body, and tail). The tail (the left lobe) was used for histopathological examination, the body part for inflammatory biomarker detection using a phosphate buffer saline, and the head (left lobe) of the specimen for RT-PCR mRNA quantification. Rats were euthanized via decapitation while under anaesthesia.

### 2.4. Biochemical Parameters

#### 2.4.1. Evaluation of Amylase Activity, Insulin, and Glucose Levels

Quantitative determination of rat serum α-amylase activity was performed utilizing a Vitro amylase reagent purchased from Vitro Scient, Belbis, Egypt. Enzymatic colorimetric determination of blood glucose (GOD-PAP methodology) was estimated using a Liqui CHEK glucose assay kit (AGAPPE. Diagnostic Ltd., Kerala, India). Serum insulin levels were assayed via a rat insulin ELISA kit (Cat. No. ERINS; Thermo Fisher Scientific, Waltham, MA, USA).

#### 2.4.2. Assessment of the Oxidative Stress Biomarkers and Inflammatory Mediators

A lipid peroxide (malondialdehyde) kit (Catalog # SD 25 29; BioDiagnostic, Giza, Egypt), a superoxide dismutase (SOD) kit (Catalog # SD 25 21; BioDiagnostic, Giza, Egypt), and a total antioxidant capacity (TAC) kit (Catalog # SD 25 13; BioDiagnostic, Giza, Egypt) were used to quantitatively detect the levels of malondialdehyde (MDA), SOD, and TAC, respectively, in the pancreatic tissue samples (colorimetric method). The Rat IL-1β Quantikine ELISA Kit (Catalog # RLB00) and Rat TNF-alpha Quantikine ELISA Kit (Catalog #: RTA00) purchased from R&D Systems, Minneapolis, MN, USA, were used to measure the levels of IL-1β, inflammatory mediators, and TNF-α.

### 2.5. Real-Time Quantitative PCR (qPCR) Analysis for ATF6, CHOP, XBP1, IRE-1α, BAX, and Casp-3 mRNA Expression

#### 2.5.1. RNA Purification and cDNA Synthesis

Trizol (Invitrogen; Thermo Fisher Scientific, Waltham, MA, USA.) was used to extract the total RNA from pancreatic tissue samples, and the A260/A280 ratio and quality of RNA were determined and analysed via a NanoDrop^®^ ND–1000 Spectrophotometer (NanoDrop Technologies; Wilmington, DE, USA). Estimated RNA purity was between 1.8 and 2.0. For cDNA synthesis, a High-Capacity cDNA Reverse Transcription Kit cDNA Kit; (Applied Biosystems™, Thermo Fisher Scientific, Waltham, MA, USA) was used.

#### 2.5.2. mRNA Quantification

Real-time polymerase chain reaction (RT-PCR) was performed using a Mx3005P Real-Time PCR System (Agilent Stratagene, Santa Clara, CA, USA) and TOPreal™ qPCR 2X PreMIX (SYBR Green with low ROX) obtained from Enzynomics, Daejeon, Korea. To initiate the PCR cycling conditions, the samples were initially denatured at 95 °C for 12 min, followed by 40 cycles of denaturation at 95 °C for 20 s, annealing at 60 °C for 30 s, and extension at 72 °C for 30 s. The oligonucleotide-specific primers were synthesized by Sangon Biotech (Beijing, China). The expression levels of the target gene were normalized using the mRNA expression of β-actin. The results were presented as fold changes using the 2^−ΔΔCT^ method [28] (Table 1).

### 2.6. Histopathologic Study

Samples of pancreatic tissue were fixed in 10% neutral-buffered formalin for 72 h and then processed in serial upgrades of ethanol and subsequently cleared in xylene. Samples were then infiltrated and embedded into the Paraplast tissue embedding media. Sections approximately 5 μm thick were sliced via a rotatory microtome to demonstrate the pancreatic parenchymal morphology following haematoxylin and eosin staining in accordance with the Bancroft and Layton 2018 protocol for routine histological examination [29].

One slide was prepared for each sample. Six representative non-overlapping fields were randomly selected and scanned from each sample by two histological professionals to study the histopathological changes.

#### 2.6.1. Immunohistochemical Study

Approximately 5 μm thick tissue sections were treated using 3% H_2_O_2_ for 20 min, rinsed, and incubated using anti-insulin (bs-0056R—1:200—Bioss Co., Woburn, MA, USA), anti-NLRP3 (GTX00763—1:100—Genetex Co., Irvine, CA, USA), and anti-Caspase-1 antibody [NB100-56565—1:100—Novusbio Co., Centennial, CO, USA] at four degrees Celsius overnight. The sections were rinsed using PBS and incubated with a secondary antibody HRP Envision kit (DAKO) for 20 min. Sections were then incubated with diaminobenzidine (DAB) for 10 min following another wash also using PBS. The samples were washed for the last time, also using PBS, and then stained with haematoxylin, dehydrated, and cleared in xylene before being covered and slipped for examination. All standard procedures were carried out according to the standard protocol for routine histological examination [29].

#### 2.6.2. IHC Analysis

One slide was prepared for each sample. Six representative non-overlapping fields were randomly selected and scanned per tissue section. The relative area percentage of positive reactions for Caspase 1, NLRP3, and anti-insulin in pancreatic islets (using the entire pancreatic parenchyma) was assessed as a standard module for IHC expressions. The true positive immune expression (indicated by brown colour) was identified in each field/tissue sample. All light microscopic examinations and data were collected using an automated-grade, standard-unit Leica Application module, which was pre-programmed for histological analysis. The module was connected to a Full HD microscopic imaging system (Leica Microsystems GmbH, Wetzlar, Germany) to analyse the total sample section based on the entire field area. Negative control staining was performed after exclusion of the primary antibody. The positive reaction was defined with a cytoplasmic brown colour.

Pancreatic tissue samples were sliced into small (1 mm^3^) pieces and fixed in 2.5% glutaraldehyde buffered to pH 7.4 with phosphate buffer for a duration of two hours. Using the same buffer, samples were washed and subsequently post-fixated via osmium tetroxide with phosphate buffer (1%) for two hours at room temperature. Using graded concentrations of ethanol, the tissue samples were dehydrated and embedded into Epson–Araldite resin. Samples were cut into ultrathin sections, then mounted on copper grids and double-stained with uranyl acetate followed by lead citrate. Using a transmission electron microscope (J.E.O.L., JEM-2100; Tokyo, Japan) operated at 80 kV, electron micrographs were obtained, and this part of the experiment was performed at the electron Microscopic Unit, Faculty of Agriculture, Mansoura University, Mansoura, Egypt.

#### 2.6.3. Computer-Assisted Digital Image Analysis (Digital Morphometric Study)

EM images were acquired and analysed using VideoTest Morphology^®^ software 5.2 on an Intel^®^ Core I7^®^-based computer. The software facilitated the extraction of the following data: area measurements, colour intensity, and object counting. Granules were automatically identified and measured based on their colour difference, size, and circularity filter. The count and area measurements were performed with calibration to the image scale bar. The intensity of mitochondria was assessed through a semi-automated routine involving a combination of automatic selection and visual assessment. Six representative non-overlapping fields were randomly chosen and scanned. The selected areas were then subjected to an intensity measurement procedure.

### 2.7. In Silico Study

Molecular docking was performed to assess the affinity of imidacloprid towards the Nrf2/Keap1 complex, PTEN for ROS/mitochondrial stress activation, and lycopene against the CHOP/IRE1-α/NLRP3 pathway. The target proteins (codes: 6QME, 5BZX, 1NWQ, 6XDB, and 7ALV) were obtained from the protein data bank. Initially, water molecules were removed from the complexes, and preparation options were utilized to correct crystallographic disorders and unfilled valence atoms. The protein structures were subjected to energy minimization using CHARMM force fields while preparing the pockets for the docking process. Using Chem-Bio Draw Ultra17.0, 2D structures of the tested compounds were drawn and saved as SDF files. The saved files were then opened, and the 3D structures were protonated. Energy minimization was carried out using the MMFF94 force field, aiming for a 0.1 RMSD kcal/mole energy. The minimized structures were prepared for docking using ligand preparation tools. The docking process was performed using MOE 2014 software [30]. The receptor was held rigid, while the ligands were allowed to be flexible. During refinement, each molecule generated twenty different poses with the proteins. The docking scores (affinity energy) of the best-fitted poses with the active sites were recorded, and 3D figures were generated using the Discovery Studio 2016 visualizer [31].

### 2.8. Statistical Analysis

Data were collected in an Excel spreadsheet and analysed using SPSS version 22. The normality distribution was assessed using both the Kolmogorov–Smirnova test and the Shapiro–Wilk test. The Kruskal–Wallis test was applied to compare different groups. Data are represented as medians (IQR), and a significance level of 5% was considered. Box and whisker plots and line graphs were used to represent the data points. Line graphs were created using Microsoft Excel 365. Both principal component analysis (PCA) and the correlation matrix were conducted using GraphPad Prism software, version 9.5.1 (GraphPad Software, San Diego, CA, USA).

## 3. Results

Upon thorough examination, it was observed that the animals maintained normal body weight gain throughout the study, exhibiting regular feeding patterns and activity levels in all groups.

### 3.1. Effect of LYC on Amylase Activity, Insulin, and Glucose Levels in IMI-Intoxicated Rats

**Figure 1:**
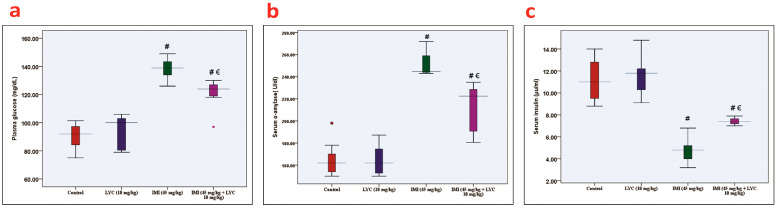
The effect of LYC on blood glucose, serum insulin, and amylase levels in rats intoxicated with IMI. (**a**): Plasma glucose (H value = 17.69, DF = 3, *p* = 0.001), (**b**): serum amylase (H value = 19.51, DF = 3, *p* = 0.000) and (**c**): serum insulin (H value = 20.07, DF = 3, *p* = 0.000). The data are represented by medians (IQR) (n = 7) at a 5% significance level (€: significant difference with IMI, #: significant difference with control).

As demonstrated in Figure 1, when tissues were exposed to toxic levels of IMI, this resulted in significantly (*p* < 0.05) elevated plasma glucose and serum amylase levels compared to the LYC and normal control groups. Serum insulin levels were significantly decreased. The administration of LYC in IMI-intoxicated rats significantly reduced the plasma glucose (*p* = 0.001) and serum amylase levels (*p* = 0.000), while the serum insulin levels were significantly increased (*p* = 0.000) similar to the IMI-intoxicated group.

### 3.2. Effect of LYC on the Oxidative Stress Biomarkers and Inflammatory Mediators in IMI-Intoxicated Rats

IMI exposure resulted in elevated (*p* = 0.001) MDA levels and reduced SOD (*p* = 0.001) and TAC (*p* = 0.000) levels in pancreatic tissues.

**Figure 2:**
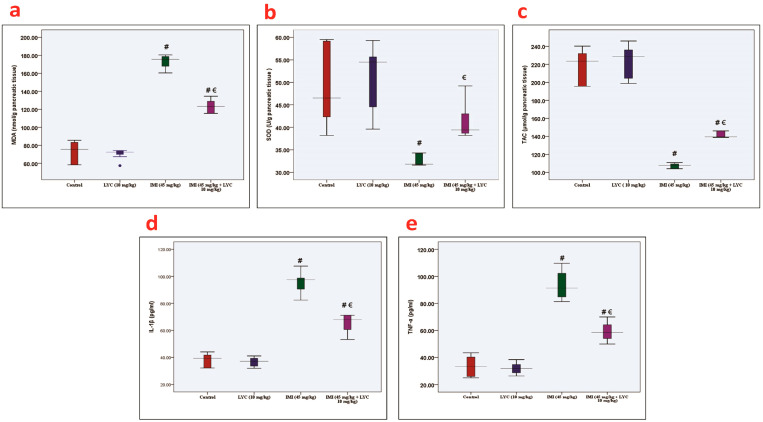
The effect of LYC on the oxidative stress biomarkers and inflammatory mediators in rats intoxicated with IMI. (**a**): MDA, (**b**): SOD, (**c**): TAC, (**d**): IL-1β, and (**e**): TNF-α. The data are represented by medians (IQR) (n = 7) at a 5% significance level. (€: significant difference with IMI, #: significant difference with control). MDA, malondialdehyde (H value = 20.54, DF = 3, *p* = 0.001); SOD, superoxide dismutase (H value = 15.67, DF = 3, *p* = 0.001); TAC, total antioxidant capacity (H value = 21.36, DF = 3, *p* = 0.000); IL-1β, interleukin 1 beta (H value = 19.90, DF = 3, *p* = 0.000); TNF-α, tumour necrosis factor-alpha (H value = 19.51, DF = 3, *p* = 0.000).

LYC administration caused a significant increase (*p* = 0.001) in SOD, and TAC (*p* = 0.000) was associated with significantly decreased (*p* = 0.001) MDA levels in the IMI+ LYC-treated group when compared with the IMI-treated group (Figure 2a–c). As shown in Figure 2d,e, IL-1β and TNF-α were significantly elevated (*p* = 0.000) in the IMI-exposed group when compared to the control and LYC groups, while LYC administration significantly reduced them in the IMI + LYC-treated group.

### 3.3. Effect of LYC on IRE-1α, ATF6, XBP1, CHOP, BAX, and Casp-3 mRNA Expression in IMI-Intoxicated Rats

**Figure 3:**
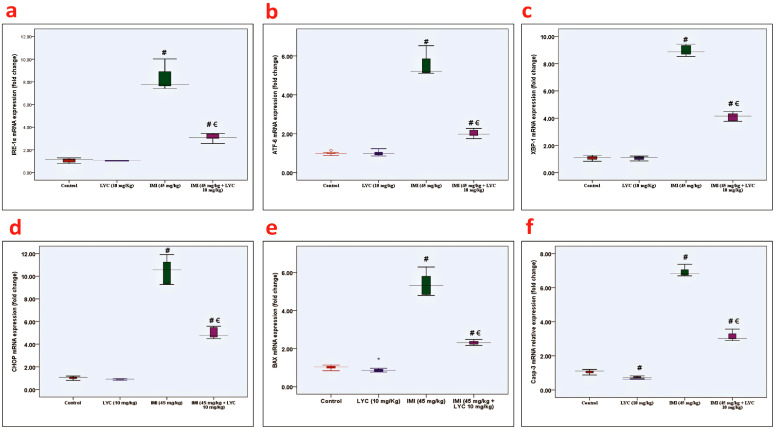
The effect of LYC on IRE-1α, ATF6, XBP1, CHOP, BAX, and Casp-3 mRNA expression in IMI-intoxicated rats. (**a**): IRE-1α (H value = 23.01, DF = 3, *p* = 0.000), (**b**): ATF6 (H value = 23.07, DF = 3, *p* = 0.000), (**c**): XBP1 (H value = 22.96, DF = 3, *p* = 0.000), (**d**): CHOP (H value = 23.60, DF = 3, *p* = 0.000), (**e**): BAX (H value = 24.61, DF = 3, *p* = 0.000), (**f**): Caspase-3 (H value = 25.38, DF = 3, *p* = 0.000). The data are represented by medians (IQR) (n = 6) at a 5% significance level. (€: significant difference with IMI, #: significant difference with control).

The qPCR analysis for the mRNA gene expression of IRE-1α (Figure 3a), ATF6 (Figure 3b), XBP1 (Figure 3c), and CHOP (Figure 3d) showed substantial upregulation (*p* = 0.000) in the IMI-exposed group in comparison to the control and LYC groups. Meanwhile, LYC administration decreased the mRNA expression of these genes in the IMI+ LYC-treated group. The apoptotic biomarkers, BAX (Figure 3e) and Caspase-3 (Figure 3f), were substantially upregulated (*p* = 0.000) in the IMI-exposed group compared to other groups, and LYC administration was remarkably downregulated (*p* = 0.000) in the IMI+ LYC-treated group when compared with the IMI-treated group.

### 3.4. Histopathological Examination

#### 3.4.1. Lycopene Alleviated Pathological Changes and Restored Insulin Secretion after IMI Intoxication

**Figure 4:**
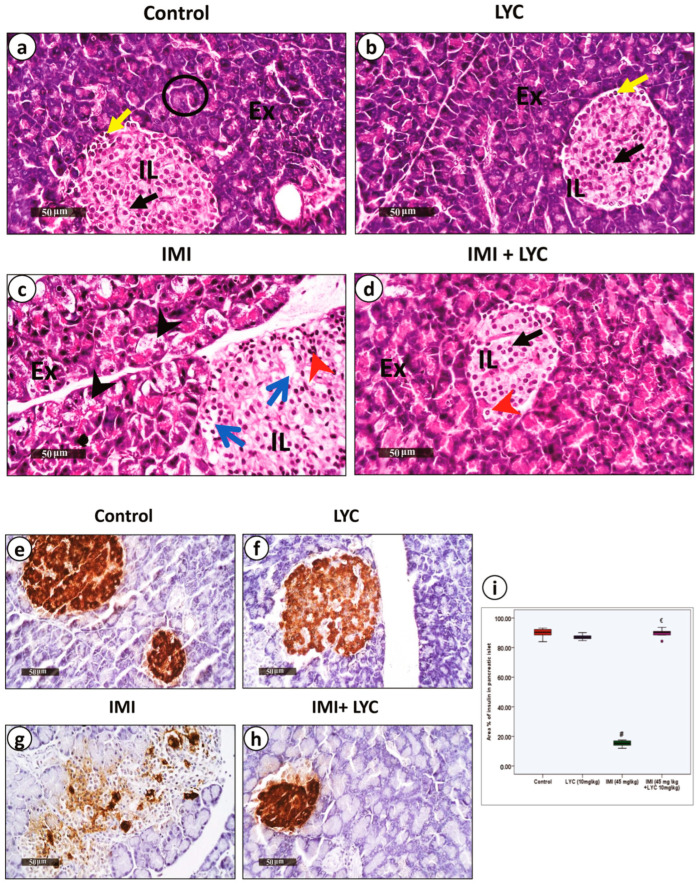
Histological pictures representing H&E and anti-insulin antibody-stained pancreatic sections from experimental groups. (**a**–**d**) H&E-stained sections. (**a**) Control group: endocrine pancreas is formed of clusters of islets of Langerhans cells (IL), which appear as pale-stained compact masses embedded within the exocrine part. The islet shows beta cells with light-stained cytoplasm and large nuclei located centrally (black arrow), while alpha cells have small, dark nuclei located at the periphery (yellow arrow). The exocrine pancreas (Ex) shows pancreatic acini (circles), and each acinar cell has rounded basal nuclei, basal basophilia, and apical acidophilia. (**b**) LYC group shows mostly the same histological arrangement as the control group with no pathological abnormalities. (**c**) IMI group shows multiple focal records of degenerated and pyknotic acinar cells with vacuolar cytoplasm (black arrowheads) accompanied by multiple clumps of degenerated beta cells with dense nuclei within the islet (red arrowheads), surrounded by vacuolar spaces (blue arrows). (**d**) IMI + LYC group shows an occasional few degenerated beta cells with apoptotic nuclei (red arrowhead), with remarkable protective efficacy on pancreatic acini resembling normal controls. (**e**–**h**) Expression of anti-insulin antibody. (**e**) Control group: strong cytoplasmic anti-insulin expression in beta cells, which occupy most of the islet. (**f**) LYC group shows nearly the same expression. (**g**) IMI group: faint anti-insulin expression indicates cessation of secretion and destructed beta cells. (**h**) IMI + LYC group shows restored ant-insulin expression. (Magnification: ×400, scale bar = 50 μm). (**i**): Histogram represents area% of insulin in pancreatic islets in all experimental groups (n = 6) (H value = 14.9, DF = 3, *p* = 0.002). (€: significant difference with IMI, #: significant difference with control).

The microscopic evaluation of H&E-stained sections (Figure 4a–d) showed that the control group, as well as the LYC (10 mg/kg) group, revealed normal parenchyma of the pancreas, including normal size and shape of the islet of Langerhans within the surrounding exocrine acini. The acinar cells appeared normal, with basal nuclei (Figure 4a,b). In contrast, rats intoxicated with IMI (45 mg/kg) had noticeable necrotic changes in both the exocrine and endocrine pancreas. The islet of Langerhans displayed degenerated cells with pyknotic nuclei. Acinar cells showed areas of degeneration, which were represented as swollen cells with vacuolar cytoplasm (Figure 4c).

After treating the intoxicated rats with LYC (10 mg/kg), there was an apparent improvement in the histopathological changes that had previously been demonstrated following IMI toxicity. However, some cells within the islets were still necrotic (Figure 4d). Insulin secretion was further studied using anti-insulin immunostaining (Figure 4e–h). Both the control group and the LYC group revealed high anti-insulin expression with no significant difference (Figure 4i). Rats intoxicated with IMI (45 mg/kg) showed significantly down-regulated anti-insulin expression (Figure 4i), indicating that the pathological changes in beta cells affected insulin secretion.

In contrast, co-administration of both IMI (45 m/kg) and LYC (10 mg/kg) significantly (*p* = 0.002) restored insulin secretion, which was demonstrated in the form of up-regulated anti-insulin expression (Figure 4i).

#### 3.4.2. Lycopene Downregulated Both Anti-NLRP3 and Anti-Caspase 1 Inflammatory Biomarkers

**Figure 5:**
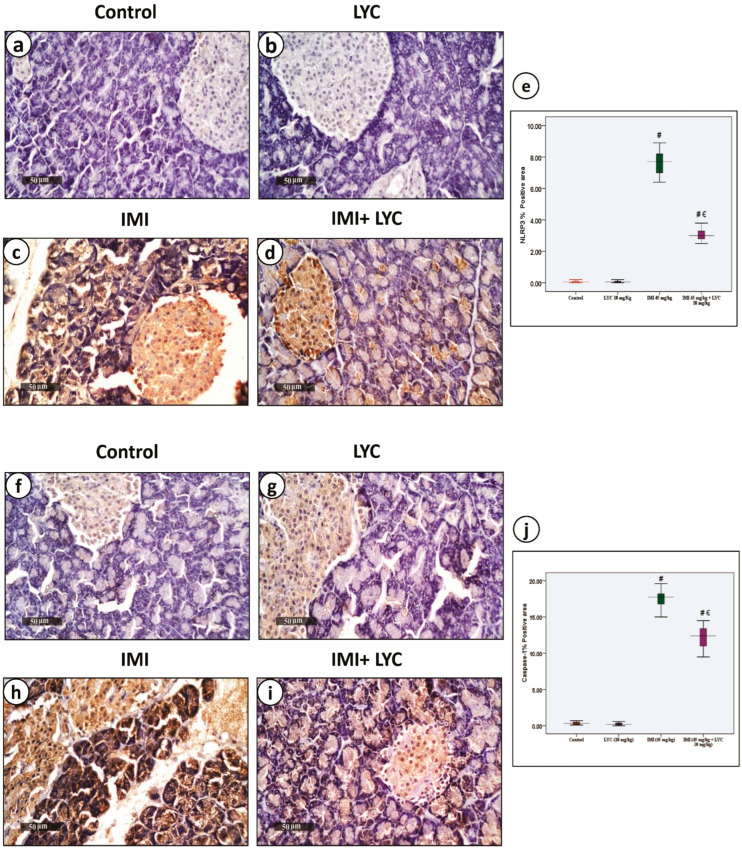
Histological pictures representing anti-NLRP3 antibody and anti-caspase 1 antibody-stained pancreatic sections from experimental groups. (**a**–**d**) Expression of anti-NLRP3 antibody. (**a**) Control group: nearly negative cytoplasmic anti-NLRP3 expression in the cells of Langerhans islets as well as exocrine acinar cells. (**b**) LYC group showed nearly the same expression as the control group. (**c**) IMI group: strong, widely expressed anti-NLRP3 in both islet cells and acinar cells indicated high inflammatory reaction. (**d**) IMI + LYC group showed mild expression of anti-NLRP3 expression, indicating cessation of inflammation. (**f**–**i**) Expression of anti-caspase 1 antibody. (**f**) Control group and (**g**) LYC group showed nearly negative cytoplasmic anti-caspase 1 immunoexpression in the cells of Langerhans islets as well as acinar cells. (**h**) IMI group: strongly upregulated anti-caspase 1 expression indicating highly inflammatory reaction. (**i**) IMI + LYC group showed cessation of anti-caspase 1 expression. Mild expression appeared in both islet cells and acinar cells. (Magnification: ×400, scale bar = 50 μm). (**e**,**j**): Histograms represent NLRP3% positive area (H value = 19.84, DF = 3, *p* = 0.000) and caspase 1% positive area (H value = 19.74, DF = 3, *p* = 0.000), respectively, in all experimental groups (n = 6). (€: significant difference with IMI, #: significant difference with control).

The immunohistochemical results of NLRP3 (Figure 5a–d) and caspase 1 (Figure 5f–i) strongly supported the histopathological findings. Pancreas samples obtained from the control group showed nearly negative anti-NLRP3 and anti-caspase 1 inflammatory biomarkers. Expression of both biomarkers in the LYC-treated group revealed that the values were almost the same as the control levels. As would be expected, rats from group III that were intoxicated with IMI (45 mg/kg) showed a significant increase in anti-NLRP3 (Figure 5e) and anti-caspase 1 (Figure 5j) expression compared to the control group. On the other hand, co-administration of both IMI (45 m/kg) and LYC (10 mg/kg) reduced inflammation. This group revealed a significant decrease (*p* = 0.000) in anti-NLRP3 and anti-caspase 1 expression, as shown in (Figure 5e,j), respectively.

#### 3.4.3. Lycopene Alleviated the Ultra-Structural Pathological Changes in Beta Cells after IMI Intoxication

**Figure 6:**
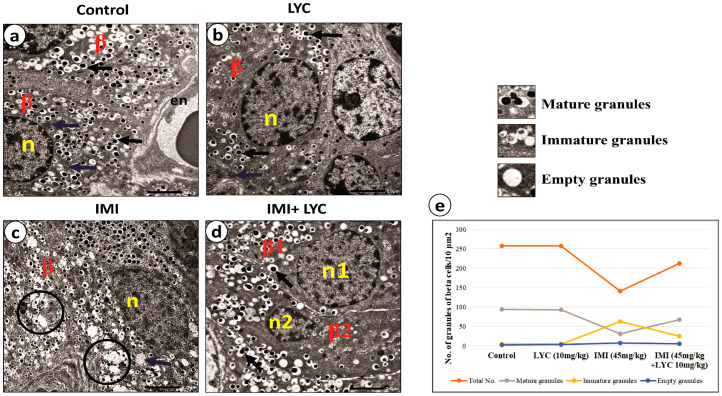
Electron micrographs representing endocrine beta cells (**a**–**d**) from experimental groups. Both (**a**) control group and (**b**) LYC group showed normal beta cells (β) with rounded vesicular nuclei (n), normal mitochondria (blue arrow), and characteristic insulin granules with dense granule cores and wide clear haloes (black arrows). N.B: part of blood sinusoid with endothelial lining (en) appeared in control group. (**c**) IMI group: beta cells showed shrunken nuclei (n). Most of the secretory granules had empty cores merging, forming large spaces (circles). The mitochondria appeared swollen, with lower density (blue arrow). (**d**) IMI + LYC group: most beta cells (β1) appeared nearly normal, with normal nuclei (n1) and insulin granules, while other beta cells (β2) appeared with shrunken nuclei (n2). (Scale bar = 2 μm). (**e**): Line graphs represent the number of beta cells granules/10 um^2^ in all experimental groups (n = 6). The granules are represented as total, mature, immature, and empty granules. Kruskal–Wallis test was applied to compare different groups. Total granules (H value = 18.19, DF = 3, *p* = 0.000), mature granules (H value = 19.47, DF = 3, *p* = 0.000), immature granules (H value = 19.52, DF = 3, *p* = 0.000), and empty granules (H value = 11.19, DF = 3, *p* = 0.011).

The most striking results in this study were the ultrastructural changes observed in beta cells (Figure 6a–d). The control group and LYC group showed nearly identical normal ultrastructures of beta cells, with euchromatic nuclei displaying prominent nucleoli and characteristic beta cell granules with dense cores surrounded by haloes and normal mitochondria (Figure 6a,b). Following intoxication with IMI, beta cells showed slightly shrunken nuclei and a large number of empty granules, while some other granules appeared with heterogeneous densities. The mitochondria appeared swollen (Figure 6c). LYC treatment of intoxicated rats showed apparent histological improvement (Figure 6d). Statistical analysis of the beta cell granule number (total, mature, immature, and empty) was performed (Figure 6e). There was a significant difference between the control and IMI groups for all variables. An apparent significant increase in immature granules (*p* = 0.000) and empty granules (*p* = 0.011) compared to mature granules was observed in the IMI group, indicating a cessation of insulin secretion. LYC treatment reversed the results of the IMI group. LYC appeared to restore the insulin secretion of beta cells. The results showed that mature granules were statistically (*p* = 0.000) the most frequent in the control and LYC groups (Figure 6e).There was a significant difference between the IMI and IMI +LYC groups for all variables except the percentage of empty granules. These results are in parallel with the biochemical results.

#### 3.4.4. Lycopene Alleviated the Ultra-Structural Pathological Changes in Pancreatic Acinar Cells after IMI Intoxication

**Figure 7:**
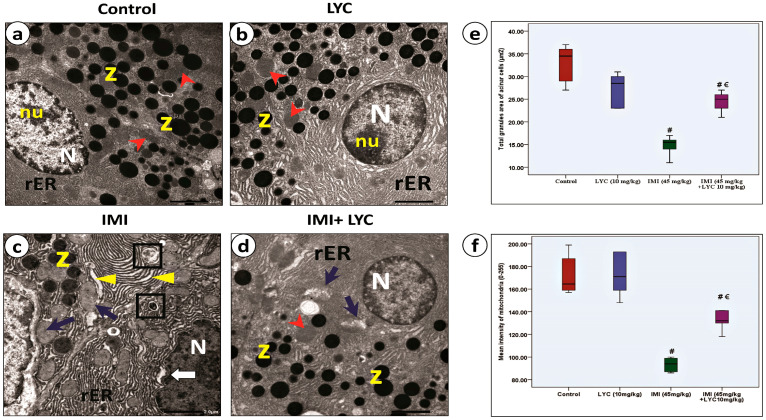
Electron micrographs representing pancreatic acinar cells (**a**–**d**) from experimental groups. (**a**) Control group showed exocrine acinar cells with large nuclei (N) displaying nucleolus (nu). The cytoplasm were packed with characteristic cisternae of rough endoplasmic reticulum (rER), large zymogen granules (z), and homogenous mitochondria (red arrowheads). (**b**) LYC group showed the same histological features as the control group. (**c**) IMI group: obvious degenerative features of pyroptosis; shrunken, non-fragmented nucleus with increased density of chromatin (N) surrounded by irregular nuclear envelope (white arrow); dilated rER cisternae (triangles); few zymogen granules (z) appeared with heterogenous density; irregularly shaped, swollen mitochondria (blue arrows); and auto-lysosomes (squares) were noticed. (**d**) IMI + LYC group: improved histological features after LYC treatment; restored zymogen granules (z) and nearly normal rER appearance. Some mitochondria appeared normal (red arrowheads), while some others are still distorted (blue arrows). (Scale bar = 2 μm). (**e**,**f**): Histograms represent total granule area (μm^2^) in acinar cells (H value = 18.19, DF = 3, *p* = 0.000) and mean intensity of mitochondria (0–255), respectively (H value = 19.14, DF = 3, *p* = 0.000). Kruskal–Wallis test was applied to compare different groups (€: significant difference with IMI, **#** significant difference with control).

Electron microscopic evaluation of pancreatic acinar cells (Figure 7a–d) showed normal structures in the control group. The cells were easily recognized by their rounded nuclei, normal mitochondria, abundant rER, and characteristic electron-dense zymogen granules. Most granules were homogenous in consistency (Figure 7a). The LYC group showed nearly the same ultrastructure, without any pathological alteration (Figure 7b). After IMI intoxication (Figure 7c), there were dramatic pyroptotic changes demonstrated in the form of shrunken irregular non-fragmented nuclei; dilated cisternae of rER; statistically decreased (*p* = 0.000) zymogen granules (μm^2^), which appeared heterogeneous in consistency (Figure 7e); and statistically decreased (*p* = 0.000) the mean intensity of the swollen mitochondria (Figure 7f). LYC reversed most of these pathological changes (Figure 7d). The ultrastructure of acinar cells apparently improved, which was determined by the presence of normal nuclei and normal rER. Some mitochondria were still heterogeneous in consistency. Moreover, both the total area of zymogen granules (μm^2^) and the mean intensity of the mitochondria were significantly increased (*p* = 0.000) (Figure 7e,f).

### 3.5. Molecular Docking Analysis

#### 3.5.1. Imidaclopride against Nrf2/Keap1 Complex and PTEN Target Proteins

**Figure 8:**
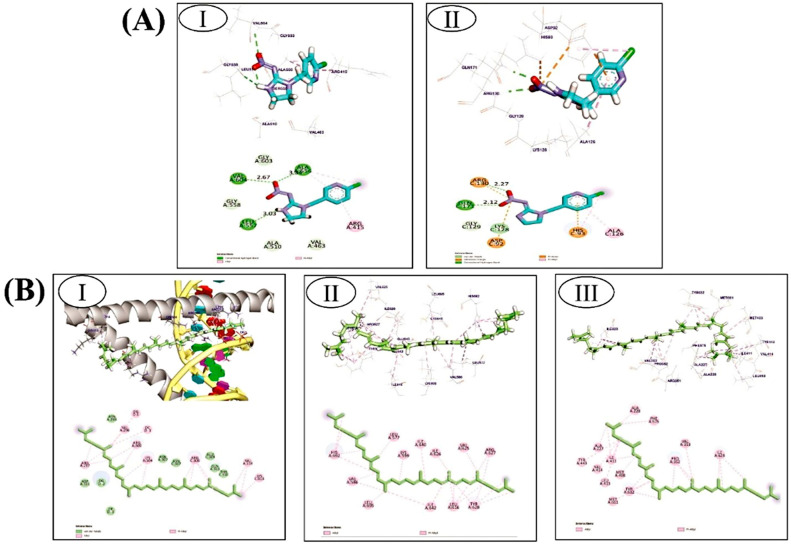
Molecular docking: (**A**(**I**)) 3D and 2D figures of imidaclopride against Nrf2/Keap1 complex; (**A**(**II**)) 3D and 2D figures of imidaclopride against PTEN; (**B**(**I**)) 3D and 2D figures of lycopene against CHOP; (**B**(**II**)) 3D and 2D figures of lycopene against IRE1-α; (**B**(**III**)) 3D and 2D figures of lycopene against NLRP3.

The binding mode of imidaclopride against the Nrf2/Keap1 complex and PTEN exhibited binding energies equal to −5.97 and −5.63 kcal/mol, respectively. imidaclopride interacted with the Nrf2/Keap1 complex and formed three hydrophobic π-Alkyl interactions with Ala556 and Arg415, which additionally formed three hydrogen bonds with Leu557, Val604, and Ala556 with distances of 3.03, 2.67, and 3.34 Å, respectively (Figure 8A(I)). On the other hand, imidaclopride interacted with His93 and Ala126 via three hydrophobic π-interactions against PTEN. Additionally, imidaclopride was observed to form two hydrogen bonds and two attractive interactions with Arg130, Asp92, and Gln171, with distances of 2.27 and 2.12 Å (Figure 8A(II)).

#### 3.5.2. Lycopene against CHOP/IRE1-α/NLRP3 Pathway

The binding mode of lycopene against CHOP, IRE1-α, and NLRP3 exhibited binding energies equal to −7.74, −8.28, and −7.83 kcal/mol, respectively. Lycopene with CHOP formed seventeen hydrophobic π-Alkyl interactions with DG1, DC1, Arg297, Val296, Arg300, Lys304, Arg306, Val314, and Lys313 (Figure 8B(I)). Additionally, lycopene interacted with His692, Val586, Leu695, Ile692, Leu616, Tyr628, Arg627, Val625, Ile626, Ile640, Lys599, and Leu577 via twenty-four hydrophobic π-Alkyl interactions against IRE1-α (Figure 8B(II)). Moreover, lycopene interacted with NLRP3 via twenty-one hydrophobic π-alkyl interactions with Ile623, Val353, Pro352, Phe575, Ala228, Ala227, Thr443, Ile411, Val414, Met408, Leu413, Tyr632, and Met667 (Figure 8B(III)).

### 3.6. Principal Component Analysis

**Figure 9:**
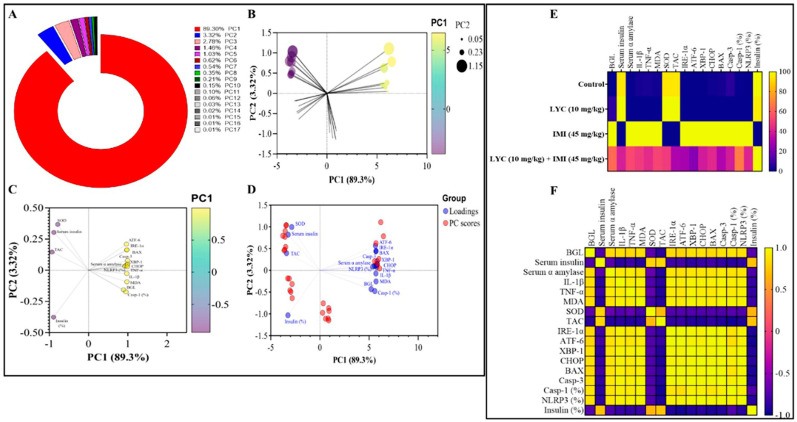
A summary of the effects of lycopene administration in IMI-induced pancreatic dysfunction. Principal component analysis (PCA) was conducted to analyse the variables (**A**–**D**). (**A**) The contribution of major components to overall variance was determined. (**B**) A PC score plot was generated, illustrating the dimensions of the different groupings based on the PC1 scale. (**C**) A PC loading plot was created, showcasing the dimensions of the studied variables according to the PC1 scale. (**D**) A PC biplot was generated, overlaying the PC scores and PC loading plots. A heatmap and correlation matrix were constructed to provide an overview of the effects of lycopene administration in IMI-induced pancreatic damage (**E**,**F**). (**E**) The heatmap displayed the studied parameters, indicating the impact of lycopene administration on IMI-induced pancreatic damage. (**F**) The correlation matrix examined the relationships between all analysed parameters.

All variables under study were loaded into two primary components (PC), PC1 and PC2, and then Primary Component analysis (PCA) was applied. PC1 and PC2 accounted for 92.63% of the total variance. Figure 9A illustrates that PC1, which accounted for the largest part of variation (89.3%), represents all parameters. In contrast, PC2 significantly reduced the variance proportion (3.32%) compared to PC1. The control and LYC groups showed antagonistic correlation with PC1, which accounted for the majority of the total variation in the PC score plot. The IMI group, on the other hand, had a favourable interaction with PC1 (Figure 9B). The control and LYC groups showed significant correlations with SOD, serum insulin level, TAC, and area of insulin-secreting cells in the pancreatic tissue, as shown in the PCA loading plot. On the other hand, the IMI group was found to be associated with the glucose level, serum α amylase level, ATF-6, IRE-1α, BAX, Casp-3, XBP-1, CHOP, TNF-α, IL-1β, MDA, and Casp-1 and NLRP3 areas in the pancreatic tissue (Figure 9C). The PCA biplot is a combination of loading and PC score plots (Figure 9D). The interchanges between all assessed variables regarding the effects of the administration of LYC in either IMI-treated or non-treated rats were described and correlated using a heatmap (Figure 9F) and a correlation matrix (Figure 9F) for all the tested parameters.

In the subsequent discussion, these findings will be further elaborated upon.

## 4. Discussion

Both oxidative stress and inflammatory cascades are significant mitigating factors that promote the development of pancreatitis. Environmental pollution exposure is a major source of health risks worldwide, especially toxic levels of pesticides. IMI is one of the most broadly used pesticides in the world [9]. IMI can exaggerate adverse effects on various body tissues, which may be fatal [10].

Based on pharmacokinetic studies indicating rapid oral absorption of IMI in mammalian systems and the subsequent distribution of its residues, it is noteworthy that the pancreas predominantly accumulates 4-hydroxy-IMI. This suggests a potential for bioaccumulation of IMI in the pancreas, which could have implications for pancreatic health and function [11].

Lycopene is a red carotenoid hydrocarbon found in various red produce, including tomatoes, red carrots, watermelons, grapefruits, and papaya. This hydrocarbon is renowned for its antioxidant and anti-inflammatory properties [32,33]. Our study is the first in the literature to investigate and report on the protective mechanisms of lycopene against IMI-induced oxidative and inflammatory damage in the pancreas using a rat model.

To create the IMI pancreatic injury rat model, IMI (45 mg/kg) was administered for 30 days. The pancreas was visibly damaged by IMI, as evidenced by the increase in pancreatic amylase activity. Similar findings of increased serum amylase activity have been observed in other studies that have examined IMI toxicity. Amylase is a marker used to detect the presence of pancreatitis. IMI interacts with the sulphydryl group present in tissue membrane proteins [34]. Consequently, the oxidative interaction of IMI may have led to the degradation of pancreatic cell membranes, causing an elevated release of pancreatic enzymes into the bloodstream. Additionally, our study revealed a significant decrease in serum insulin levels and a corresponding increase in blood glucose levels, consistent with findings by Khalil et al., which can be attributed to IMI impairing downstream targets of glucose transporter 4 translocations in myotubes and adipocytes through the disruption of insulin receptor substrate-1 activation via calcium-dependent mechanisms [35,36].

Furthermore, IMI induces oxidative stress and inflammation, which have been proposed as the underlying causes of hyperglycemia and decreased insulin levels, as suggested by Sun et al. (2021) and Sun et al. (2016). Our study focused on the damage inflicted on β cells by IMI, as observed through microscopy, which revealed smaller nuclei and depleted secretory granules. These findings provide evidence that pancreatic β cells, with reduced levels of antioxidant enzymes, are consistently targeted by IMI accumulation. This is further supported by the detection of diminished anti-insulin expression in pancreatic sections stained with an anti-insulin antibody, indicating a cessation of insulin production and confirming the destruction of β cells in rats exposed to IMI alone. Our study demonstrated the vulnerability of β cells to IMI-induced reactive oxygen species (ROS), resulting in reduced insulin production and elevated blood glucose levels. However, the administration of lycopene reversed these detrimental effects [32]. Similarly, our study demonstrated that lycopene reduced amylase levels and blood glucose while increasing plasma insulin values [37]. Lycopene protected β cells from oxidative damage, preserved the integrity of the cell membrane against IMI toxicity, and mitigated ROS-induced cellular damage. Excess ROS from IMI exposure leads to oxidative lipid, protein, and DNA damage as well as cell death, all of which are mitigating factors for common diseases [12]. This study also investigated the pancreatic tissue levels of antioxidant enzymes SOD and TAC, as well as biomarkers for lipid peroxidation, like MDA. IMI exposure in group II exhibited a significant increase in the pancreatic level of MDA, which was coupled with a significant decrease in the pancreatic levels of SOD and TAC when compared to the control group. Likewise, Saber et al. reported on dysregulated tissue redox status associated with IMI exposure [13]. IMI-induced oxidative damage resulted in increased lipid peroxidation and protein oxidation [9]. Additionally, pancreatic β cells are susceptible to oxidative stress because of their minor antioxidant capacity and high endogenous ROS production, which results in irreversible damage, mitochondrial malfunction, and subsequent apoptosis [8]. Singh et al. (2020) elucidated that LYC supplementation upregulated the expression of the genes that code for antioxidant enzymes and downregulated those for oxidation of lipids, and consequently exhibited its antioxidative capacity [38].

In reference to the inflammatory cytokine storm in pancreatic tissue that is associated with IMI exposure, IMI exposure increased pancreatic levels of pro-inflammatory cytokines (TNF-α and IL-1β), a finding reported by Duzguner and Erdogan (2012) [39]. This could be explained by the fact that oxidative stress and overproduction of ROS induce cellular damage via membrane phospholipid peroxidation and cytoskeletal protein degradation, leading to the activation of proinflammatory cytokines and initiating inflammatory response [40]. Furthermore, IMI exposure in group II revealed a significant increase in serum amylase enzyme. This confirmed that pancreatitis can be caused by excess exposure of IMI, a finding reported by Khandia et al. (2020) [41]. Interestingly, LYC supplementation with IMI could inhibit proinflammatory cytokine and chemokine expression and decrease serum amylase levels [14,42]. This could be explained by the fact that LYC inhibits the NF-κB signalling pathway by reducing the production of ROS and inhibiting pro-inflammatory cytokine release [15], thereby improving IMI-induced pancreatitis [21]. Lycopene supplementation opposed the IMI-induced pancreatic islet injury and enhanced serum insulin levels. It effectively opposed the rise in ROS and pro-inflammatory cytokine levels, thereby maintaining the architecture of pancreatic islets [38].

ER stress is crucial in the development and pathophysiology of several illnesses, including damage to the pancreas. It is well established that the endocrine and exocrine arms of the pancreas are susceptible to catastrophic consequences from chronic ER stress. Notably, the exocrine component and pancreatic B cells experience increased ER stress due to oxidative stress, inflammation, and lipo-toxicity. The natural, transient, adaptive, and defensive processes that lessen ER stress and guarantee proper protein folding are fought by these triggering stimuli [13]. Nevertheless, over time, the trigger chaperones play a role in ER protein folding, and as ER stress develops, the ER’s capacity to fold proteins becomes unable to manage many unfolded and misfolded proteins. Consequently, ER homeostasis cannot be restored [8]. Misfolded proteins, particularly in the β cells and throughout the exocrine component of the pancreas, lead to malfunction in these regions.

The ER is where insulin production starts, and it is there that proinsulin folds appropriately to facilitate its efficient export from the ER to the Golgi, where it is packed and then cleaved to create mature insulin. As a result, the release of mature insulin decreases. Additionally, these misfolded proinsulin particles accumulate within the ER of pancreatic β cells, subsequently activating ER stress-associated pathways that mitigate more ER stress and further apoptosis of the β cells of the pancreas [5]. Mechanistically, activating transmembrane proteins, including Inositol-Requiring Enzyme 1 Alpha (IRE1α) and Activating Transcription Factor 6 (ATF6), are involved in the signal transduction of ER stress. These proteins are inactive because they are linked to the luminal binding protein (BiP) under typical circumstances [4]. During cellular stress, BiP is activated and chaperones the folding of unfolded or misfolded proteins in the ER lumen, which triggers IRE1α and ATF6 signalling cascade. IRE1α is a transmembrane protein with (1) a cytoplasmic domain that has intrinsic kinase and ribonuclease (RNase) activity and (2) an ER luminal domain designed to detect unfolded or misfolded proteins. IRE1α RNase activity strips 26 nucleotides from the X-box binding protein 1 (XBP1) mRNA [4]. This un-spliced mRNA is then converted to shorter spliced XBP1 (XBP1s), which is subsequently translated to the XBP1s transcription factor, causing the transcription of more ER stress genes [5]. Additionally, IRE1 delivers either adaptive or death signals via endoribonuclease activity, which is regulated through IRE1-dependent decay of mRNA (RIDD. Similarly, ATF6 also has both an ER luminal and a cytoplasmic domain which senses the protein folding condition. The cytoplasmic domain functions as a transcription factor regulating XBP1 expression and genes associated with ER stress, including [4] C/EBP homologous protein (CHOP), which activates apoptosis [17].

Our data reveal that ER stress is mediated by the following: (1) there is a significant increase in pancreatic IRE1α mRNA, which is related to the ER stress-associated inflammatory response, and (2) there is a marked elevation of pancreatic XBP1 and ATF6, as well as the initiator of apoptosis, CHOP mRNA. EM examination of the pancreas confirmed that the ER stress observed in IMI-treated rats was in the form of dilatation and disruption of ER. This data are similar to the results demonstrated by (1) Wu et al., 2021, who demonstrated that cadmium induces endoplasmic reticulum stress in the pig pancreas; (2) Zhang et al., 2020, who reported on its effects on the pancreas in a type 2 diabetic model; and (3) Hu et al., 2021, who studied H_2_O_2_-induced pancreatic damage [43,44,45].

It has been demonstrated that IMI not only induces oxidative damage, but also acts as a mutagen, modifying gene expression and impeding cell death. The process of apoptosis, controlled cell death, is regulated by proteins known as pro- and anti-apoptotic factors. Changes in gene expression, DNA damage, and oxidative stress are contributing factors to intrinsic apoptosis. Exposure to IMI leads to in vitro cytotoxicity and genotoxicity, resulting in molecular and DNA damage. Evidence suggests that endoplasmic reticulum (ER) stress may play a role in apoptosis. As previously discussed, CHOP (C/EBP homologous protein) acts as a critical regulator of ER stress-induced apoptosis [39]. CHOP initiates programmed cell death when ER homeostasis cannot be restored, as indicated by Hu et al. (2019), by participating in apoptotic signalling pathways. ER stress activates CHOP, a transcription factor involved in pro-apoptotic responses [46]. According to Adali and Erbaş (2023), CHOP may contribute to the pathogenesis of certain diseases by promoting cell death in cases of severe or prolonged ER stress. CHOP upregulates the expression of death receptors 4 and 5 (DR4, 5) and interacts with the death receptor pathway, ultimately leading to apoptosis [47]. Subsequently, the expression of BAX, a pro-apoptotic protein, is upregulated, while anti-apoptotic proteins are downregulated. In addition, CHOP expression inhibits Akt phosphorylation. Akt plays a significant role in cellular signalling pathways involved in metabolism, growth, division, and the inhibition of apoptosis. Akt suppression by CHOP affects the activity of caspase-3, a death protease that cleaves various important cellular proteins [46,48].

The current study revealed that IMI-exposed pancreatic tissue of rats resulted in a significant upregulation of apoptotic biomarkers, BAX, and Caspase 3 mRNA expression, which is consistent with the results of other studies [21,22].

Numerous studies have documented that ER stress, pyroptosis, and apoptosis are interdependent [20,24]. The ER stores most of the cell’s calcium, which, in a state of ER stress, activates calcium-sensing receptors and subsequently activates the NLRP3 inflammasome [6,20]. NLRP3 detects high ROS concentrations, which encourages the NLRP3 inflammasome’s development and activation. Furthermore, TNF-α is essential for the activation of caspase-1 [27]. All of these factors can enhance pyroptosis.

In this study, pyroptosis mediators were analysed in order to better understand the connection between pyroptosis and IMI-induced pancreatitis. It was shown that IMI upregulated pancreatic immunohistochemical expression of NLRP3 and elevated the levels of caspase-1 in rats exposed to IMI alone compared to unexposed rats. Previous studies have indicated that IMI induces hepatic and renal tissue pyroptosis by increasing the expression of pyroptotic biomarkers, including NLRP3 and caspase 1, which would be consistent with our results [25,26].

**Figure 10:**
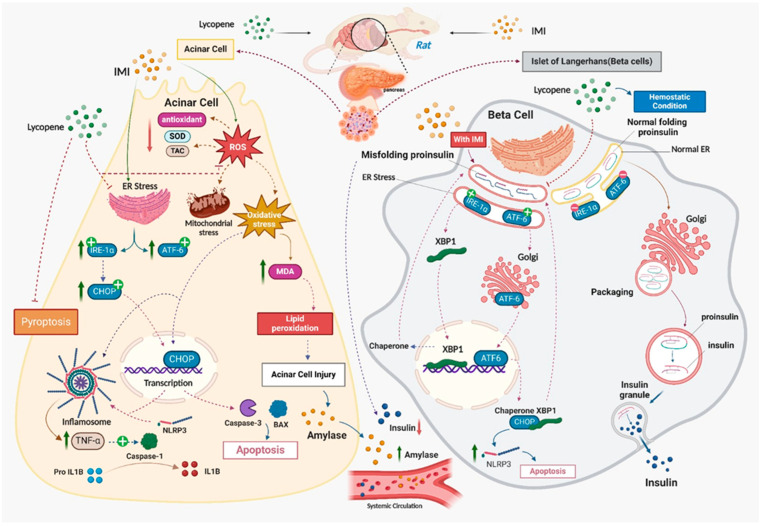
A diagrammatic scheme summarizes the proposed molecular mechanisms of the protective effect of lycopene against IMI-mediated exocrine and endocrine pancreatic dysfunction.

Numerous studies have demonstrated that supplementing with lycopene alleviates the loss of exocrine pancreatic function and β cell dysfunction, but the underlying mechanisms by which lycopene exerts these effects remain unclear. According to this study, lycopene reduced the mRNA expression of CHOP as well as the apoptotic biomarkers Cleaved-Caspase3 and Bax. Additionally, it resisted the ER stress-mediated pathway, which manifested as a substantial decline in pancreatic XBP1 and ATF6 mRNA expression. Furthermore, lycopene administration improved the suppression of immunohistochemical staining in pyroptotic indicators, such as caspase 1 and NLRP3. The data have been simplified, and the effects of LYC on IMI-induced pancreatic dysfunction in rats can be best seen using PCA. According to PCA, the administration of IMI is linked to reduced endocrine function and pancreatitis, as demonstrated by elevated glucose levels and serum α-amylase, respectively. Furthermore, increased endoplasmic reticulum stress, as demonstrated by higher expression levels of ATF-6, IRE-1α, and XBP-1, as well as elevated apoptosis, as indicated by elevated levels of Casp-3, XBP-1, and Chop, were substantially correlated with IMI treatment, as observed in PCA. Administering IMI was also related to oxidative stress and pancreatic cell inflammation, as indicated by elevated MDA and IL-1β and TNF-α levels, respectively. Furthermore, the in silico molecular docking study provides evidence of potential binding affinities between imidacloprid and lycopene and the binding pockets of target proteins, including PTEN, Nrf2/Keap1, CHOP, IRE1α, and NLRP3. The results demonstrate a significant affinity between imidacloprid and PTEN, as well as Nrf2, indicating that imidacloprid can bind to these targets and impede their protective effects, resulting in increased reactive oxygen species (ROS) levels, mitochondrial stress, and reduced antioxidant activity. In contrast, the findings indicate the promising anti-inflammatory, anti-apoptotic, and protective effects of lycopene. Lycopene exhibits the ability to suppress endoplasmic reticulum (ER) stress and decrease inflammatory mediators by blocking CHOP, IRE-1α, and NLRP3. Thus, these findings show that lycopene’s anti-apoptotic and pyroptotic properties (Figure 10), as well as the fact that it decreases ER stress, are what give lycopene its beneficial effects. According to earlier research, lycopene protects against damage to the kidneys, liver, spleen, and heart by inhibiting pyroptosis, ER stress, and pathological apoptosis, respectively. These observations are in line with our findings [28,29,30,31]. The study had some limitations, as it did not test all relevant exocrine pancreatic functions, used a single concentration of imidacloprid and lycopene without exploring dose–response relationships, and relied on a single time point for testing. Additionally, the scarcity of related studies restricts comparison. Further investigations are necessary.

## 5. Conclusions

Pyroptosis and ER stress play crucial roles in the pathogenesis of IMI-induced β cell dysfunction and loss of exocrine pancreatic function. Apoptosis serves as a critical signalling link connecting IMI-induced ER stress and pyroptosis. Oxidative stress and the inflammatory pathway also contribute to the acceleration of pancreatic damage caused by IMI toxicity. Additionally, this study provides new insights into the protective mechanisms of lycopene against IMI-induced pancreatic dysfunction. Lycopene reduces the expression of IRE1α, XBP1, and ATF6, and inhibits CHOP-mediated activation of pyroptosis and apoptosis, as shown in Figure 8. However, further investigation is needed to understand the underlying mechanisms by which IMI promotes exocrine and endocrine dysfunction of the pancreas and how lycopene protects this gland.

## Figures and Tables

**Table 1:** Primer sequences used in RT-PCR.

Gene	Forward Primer	Reverse Primer	Size	Accession No.
IRE-1α	GCGCAGGTGCAATGACATAC	CTCTTCCACGTGTGTTGGGA	178	NM_001191926.1
ATF6	AAGTGAAGAACCATTACTTTATATC	TTTCTGCTGGCTATTTGT	157	NM_001107196.1
XBP1	TTACGAGAGAAAACTCATGGGC	GGGTCCAACTTGTCCAGAATGC	289	NM_001004210.2
CHOP	CACAAGCACCTCCCAAAG	CCTGCTCCTTCTCCTTCAT	158	NM_001109986.1
BAX	CGAATTGGCGATGAACTGGA	CAAACATGTCAGCTGCCACAC	109	NM_017059.2
Casp-3	GAGACAGACAGTGGAACTGACGATG	GGCGCAAAGTGACTGGATGA	147	NM_012922.2
act-b	AACCTTCTTGCAGCTCCTCC	CCATACCCACCATCACACCC	193	NM_031144.3

## Data Availability

All data generated or analysed during this study are included in the research article.
